# Abietane Diterpenoids from the Hairy Roots of *Salvia corrugata*

**DOI:** 10.3390/molecules26175144

**Published:** 2021-08-25

**Authors:** Roméo Arago Dougué Kentsop, Valeria Iobbi, Giuliana Donadio, Barbara Ruffoni, Nunziatina De Tommasi, Angela Bisio

**Affiliations:** 1 Dipartimento di Farmacia, Università di Genova, Viale Cembrano 4, 16148 Genova, Italy; dougue.kentsop.phd@difar.unige.it (R.A.D.K.); valeria.iobbi@edu.unige.it (V.I.); 2 Consiglio per la Ricerca e la Sperimentazione in Agricoltura—CREA Centro di Ricerca Orticoltura e Florovivaismo, Corso degli Inglesi, 508, 18038 Sanremo, Italy; barbara.ruffoni@crea.gov.it; 3 Dipartimento di Farmacia, Università di Salerno, Via Giovanni Paolo II 132, 84084 Salerno, Italy; detommasi@unisa.it

**Keywords:** in vitro tissue culture, temporary immersion system (TIS), abietane diterpenoids, ferruginol, agastol

## Abstract

*Salvia corrugata* Vahl. is an interesting source of abietane and *abeo*-abietane compounds that showed antibacterial, antitumor, and cytotoxic activities. The aim of the study was to obtain transformed roots of *S. corrugata* and to evaluate the production of terpenoids in comparison with in vivo root production. Hairy roots were initiated from leaf explants by infection with ATCC 15834 *Agrobacterium rhizogenes* onto hormone-free Murashige and Skoog (MS) solid medium. Transformation was confirmed by polymerase chain reaction analysis of *rolC* and *virC1* genes. The biomass production was obtained in hormone-free liquid MS medium using Temporary Immersion System bioreactor RITA^®^. The chromatographic separation of the methanolic extract of the untransformed roots afforded horminone, ferruginol, 7-*O*-acetylhorminone and 7-*O*-methylhorminone. Agastol and ferruginol were isolated and quantified from the hairy roots. The amount of these metabolites indicated that the hairy roots of *S. corrugata* can be considered a source of these compounds.

## 1. Introduction

Hairy root culture is at present considered a convenient and efficient organ-based tissue culture system to produce bioactive secondary metabolites in a shorter time than in vivo harvesting or other in vitro techniques [1]. Abietane diterpenoids, also with rearranged skeletal types, have been described for the aerial parts and the roots of many *Salvia* species [2], mainly European and Asian species [2,3], while their presence is commonly described as limited to the roots of the New World species [4,5]. These compounds are also referred to be produced from hairy root cultures of some of these plants [4,6,7,8]. Abietane diterpenoids exhibit several interesting biological activities including antimicrobial, antifungal, anti-leishmanial, antiplasmodial, antiviral, antioxidant, antitumour, cytotoxic and anti-inflammatory activities [2,3,9,10,11,12]. Ferruginol is one of the most studied abietane diterpenoids and shows many of these important bioactivities [9,12,13,14,15,16]. The antiviral activity of this compound has been underlined [17], and interestingly against coronaviruses [18,19] i.e., SARS-CoV 3CL^pro^ [19,20]. High oxidized and rearranged abietane diterpenoids, such as *abeo*-abietane compounds, show the same biological activities [21,22,23,24,25]. Agastol, an abeo-abietane diterpenoid, showed cytotoxic activity [26], immunomodulatory effect [27] and significant inhibitory effects against human immunodeficiency virus type 1 (HIV-1) protease activity with ICs0 values of 360 µM [28]. Hairy root cultures can represent a promising source of these bioactive molecules [7] and owing to the increasing interest in natural compounds active against coronaviruses, specifically of terpenoids, in vitro culture technologies can represent efficient systems of production of secondary plant antiviral metabolites [29].

*Salvia corrugata* Vahl. is a Mexican species [30,31], used as an ornamental, characterized by abietane and *abeo*-abietane diterpenoids [32,33]. Previous work showed that the callus produced only traces of the two main icetexane diterpenoids characteristic of this species, namely fruticuline A and demethylfruticuline A, while regenerated shoot and micropropagated plant methanolic extracts contained the two icetexane as well as seven other diterpenoids, one icetexane and six abietanes [34]. The presence of these skeletons in the aerial parts of several American species has been explained, suggesting an evolutionary link between these and the Chinese ones [4,5,35]. The compounds isolated from the aerial parts and the in vitro biomass of *S. corrugata* showed antimicrobial [32,34], pro-apoptotic and pro-anoikis [36], and cancer chemopreventive [33] activities. No information is present in the literature about the presence of these compounds in the transformed hairy root biomass. The aim of the present paper was the establishment of a protocol for hairy root production and the analysis of the methanolic extract of the obtained biomass, as well as of the untransformed roots to complete the chemical analysis of the species and to evaluate the presence of abietane diterpenoids also in the hairy roots, which is the best tissue to perform the biomass scale-up in vitro.

## 2. Results

### 2.1. Establishment of Hairy Root Cultures

**Figure 1:**
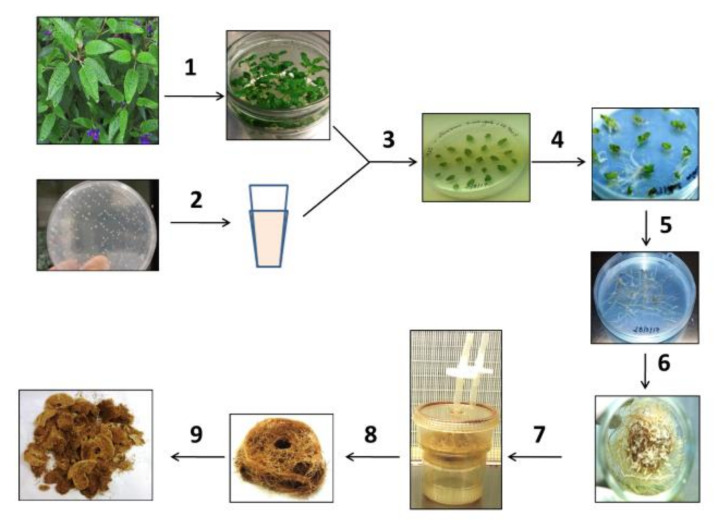
Scheme for obtaining the hairy roots of *Salvia corrugata* by transformation with *A. rhizogenes*. 1. Apical sterilization and in vitro growth, 2. suspension of *A. rhizogenes*, 3. isolation and damage of the leaves—joint incubation with *A. rhizogenes* for 20 min—co-culture on solid MS medium—after 3 days transfer on solid MS supplemented with cefotaxime, 4. formation of hairy roots, 5. clone selection, 6. growth in glass vessel containing liquid medium, 7. transfer into TIS bioreactors with constant renewal of the medium, 8. harvest of fresh biomass, 9. final biomass after lyophilization.

After the infection of *S. corrugata* explants with *A. rhizogenes* strains ATCC 15834 and LBA 9402 (Figure 1), the putative roots were observed at the wounded sites of the explants after 10 to 14 days after inoculation (Figure S1, Supplementary Materials).

After 30 days of sub-culture, the highest frequency of root induction (n. of fragment showing root development, 75%) was achieved on explants infected with strain ATCC 15834, while the hypervirulent strain LBA 9402 induced putative roots in 38% of treated fragments. A total of 14.3% of untreated fragments (control) showed root development, probably due to specific internal hormonal balance (Table 1).

Each fragment showed the development of several roots; different clones were obtained by excision and isolation of putative roots from the mother leaf explants. Twenty-six and eight clones were isolated from *A. rhizogenes* strains ATCC 15834 and LBA 9402, respectively, and one clone from the control (Table S1, Supplementary Materials). These roots were grown separately on 3 cm Petri dishes containing 3 mL solid hormone-free MS medium supplemented with cefotaxime (100 mg/L) for 30 days to investigate the daily increases in length and branching index. FA8 and FA13 clones, induced from wild type *A. rhizogenes* ATCC 15834, showed a daily increase in length of 1 mm and branching of 0.20 and 0.13, respectively (Table S2, Supplementary Materials). FL7 clone, induced from strain LBA 9402 (Table S3, Supplementary Materials), showed a maximum daily increase in length of 0.70 mm and branching of 0.33. The root clone from the control explants considerably slowed down the growth once excised from the mother explant. In fact, transferring to the new medium had a daily increase of 0.07 mm reaching 2 mm in a month and had no branches formation. However, not all the isolated putative hairy root (HR) clones of *S. corrugata* displayed both a HR phenotype and a vigorous growth. Indeed, during this period, some of them stopped their growth and died. Root viability was assessed by the observation of green fluorescence at the top of the root after calcein acetoxymethylester (AM) and fluorescein diacetate (FDA) staining. (Figure S2, Supplementary Materials). The lateral hairs were slightly reactive to calcein AM and were observed only under the bright-field option. The hairs were very well visible under both bright-field and florescent light following the FDA stain. Non-viable cells inside the root stained pale or did not show any fluorescence. Microscopical observations permitted to define a root mean diameter of 200 µm. Nadi reagent, specific for terpenoids, displayed a strong positive response (Figure S3, Supplementary Materials).

### 2.2. Confirmation of Transformation

**Figure 2:**
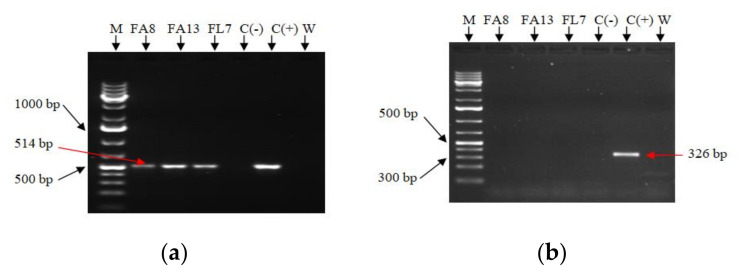
Agarose gel electrophoresis of PCR amplification of genes in putative transformed roots. (**a**) The expected 514 bp *rolC* fragment; (**b**) the expected 326 bp *virC1* fragment. M: marker 1kb plus; FA8, FA13 and FA25: respective clones of hairy root FA8, FA13 and FL7; C(−): not-transformed root (negative control); C(+): plasmid DNA of *A. rhizogenes* ATCC 15834 (positive control); W: solution without DNA.

To assess the genetic status of the hairy roots and confirm the integration of T-DNA from *A. rhizogenes* into the hairy root genome, PCR was used to target the *A. rhizogenes rolC* gene (fragment of 514 bp) located on independent T-DNAs (TL-DNA) of Ri plasmid. Three putative HR clones (FA8, FA13 and FL7) were confirmed positive to *rolC* gene by the presence of the expected 514 bp amplification band. Non-transformed root and blank solution (negative controls) were correctly detected negative (Figure 2). The eventual bacterial contamination was detected by PCR amplification of a 326 bp fragment of the *virC1* gene. The amplification band of the *virC1* gene was detected positively only in the *A. rhizogenes* sample (positive control), while all the putative HR clones, non-transformed root and the solution without DNA were detected negative to *virC1* gene. The contemporary presence of PCR amplification of 514 bp fragment of *rolC* gene and the absence of 326 bp fragment of the *virC1* gene showed that all the selected clones FA8, FA13 and FL7 contained the transgene into their genome, and they were not contaminated.

### 2.3. Growth of Hairy Root Cultures

HR clone FA8 grown in ½ WPM medium showed the best tissue production in terms of fresh weight (47.3 ± 2.0 g/L) (Figure S4, Supplementary Materials). However, the dry biomass weight in ½ WPM medium was not statistically significant compared to other culture media. Sucrose concentration had a significant influence on the growth of transformed roots. The medium containing 30 g/L sucrose showed the fastest growth (40.7 ± 4.0 g/L fresh weight; 3.7 ± 0.5 dry weight) (Figure S5, Supplementary Materials).

To set up the growth curve, the HR cultured in MS0 medium were sampled at 7-day intervals, and a typical sigmoid curve was determined after the growth kinetics assessment based on the fresh and dry weights evaluation (Figure S6, Supplementary Materials). The fresh hairy roots showed a latent phase (lag phase) in the first week, then a logarithmic phase (week 2 to 4), owing to the rapid cell division and excess of nutrients (exponential phase), and finally a plateau (week 5 to 6). After 6 weeks of culture, the hairy roots showed a dense net and a high biomass production. At this period, about 2.81 ± 0.27 g FW and 0.28 ± 0.03 g DW of hairy roots were harvested. The dry to fresh mass ratio (~10%) indicated a high accumulation of dry matter. A dark brown color at the biomass center indicated a senescent core while a clearly vital biomass was present in the external part. The medium showed a light brown color. Considering the lag phase as the more active stage of the HR culture, it is possible to assume that 21-day-old hairy roots at the intermediate growth stage were favorable for elicitation. The conductivity of the tissue culture medium which corresponds to the concentration of total components, decreased in this culture from the beginning (*t*_0_: 5.60 mS/cm) to the fifth week (4.95 mS/cm) and increased during the last week (fifth to sixth week) (5.24 mS/cm) (Figure S7, Supplementary Materials). The decrease in conductivity could be related mainly to the consumption of nutrients for growth and the production of secondary metabolites; in fact, an indirect correlation between the medium conductivity and the HR fresh weight was evidenced. The pH evolution of the medium showed a triphasic curve; a decrease from 5.12 to 4.02 from *t*_0_ to the third week, with a severe decrease during the first week; an increase to 4.56 from the third to the fifth week; a certain level of stability during the last week (Figure S7, Supplementary Materials).

The culture in the bioreactor was permitted to develop a high amount of biomass. After three months of cultivation, a non-homogeneous distribution of the tissues was recorded. The external part of the hairy root net was alive and white/yellowish in color, while the central part was dark, with a dense net clump of senescent root tissue that hampered a block of liquid medium flow and thus limited oxygen availability. The culture medium began to turn brown, a sign of aging of the hairy roots. At this time, the hairy roots were harvested, weighted, and freeze-dried. The data of the biomass produced by the different clones FA8, FA13 and FL7 are reported in Table 2. The number of bioreactors obtained for the three clones tested was markedly different (13, 4 and 1 for FA8, FA13 and FA7, respectively), as well as the total biomass, and this suggested that the materials produced were subjected to heterogeneous growth rates.

### 2.4. Phytochemical Analysis

Mass spectrometry-based analyses were performed on the extracts obtained from the three hairy root clones, to detect and quantify fruticuline A and demethylfruticuline A. Unfortunately, in all the samples analyzed the two compounds were not present, or their amount was lower than the LLOD (0.1 µg/mL). Moreover, the LC-MS/MS analysis of the methanolic extracts of the three clones did not show notable differences in phytochemical composition.

**Figure 3:**
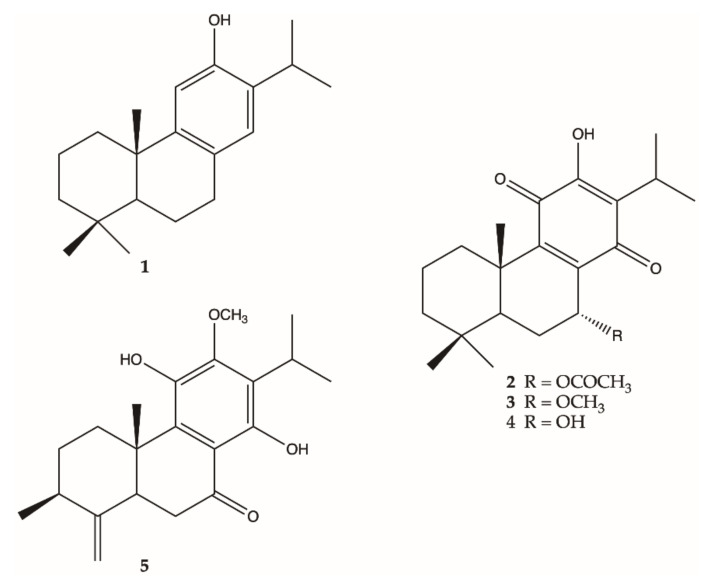
Chemical structures of abietane diterpenes isolated from the untransformed roots and hairy root of. *S. corrugata*.

The chromatographic separation of the methanolic extract of the roots of *S. corrugata* afforded four pure abietane diterpenoids, namely ferruginol (**1**) [37,38], 7-*O*-acetylhorminone (**2**) [38] and 7-*O*-methylhorminone (**3**) [39], horminone (**4**) [38,39,40,41] (Figure 3), as well as a mixture of ursolic and oleanolic acid [42].

The methanolic extract of the hairy roots showed the presence of ferruginol (**1**) [37,38], agastol (**5**) [26,43] (Figure 3), and a mixture of ursolic and oleanolic acid [42].

All compounds were identified by their physical and spectroscopic data, which were largely consistent with those published in the literature.

The quali-quantitative analysis by HPLC-DAD showed that the relative amounts of **1** and **5** (*w*/*w*, %) in the extract were 31.2 ± 3.3% and 33.6 ± 2.8%, respectively, corresponding to theoretical extractive yields of 10.8% and 11.6% from the hairy root biomass, respectively.

## 3. Discussion

The fundamental basis of an efficient hairy root culture process is the development of an appropriate hairy root clone line that maximizes both growth and rate and product yield [44]. The definition of hairy root is related to at least three aspects, two out of them are morphological: the inverse geotropism and the abundant branching in the absence of auxine in the medium, and the last is the molecular evidence of integration. The integration of the T-DNA region with its genes in the plant genome is necessary for hairy root induction [45]. A few days after co-cultivation with bacterial strain, putative hairy root arose. In this study, the percentage of root induction from *S. corrugata* leaf explants treated with the wild-type *A. rhizogenes* ATCC 15834 was almost double that obtained by the hyper-virulent strain *A. rhizogenes* LBA 9402. According to our results, the induction percentage of 75% obtained with *A. rhizogenes* ATCC15834 was relatively high compared to other *Salvia* species: 56.7% for *S. officinalis* [46], 56% for *S. bulleyana* [47], 20% for *S. miltiorrhiza* [48], and 22% for *S. wagneriana* [49]. By contrast, the co-cultivation of *S. corrugata* with the hypervirulent strain LBA 9402 induced a relatively low rate of putative HR buds (38.1%) compared to 80% reported for *S. miltiorrhiza* [48], and 100% for *Ocimum basilicum* L [50]. D’ Angiolillo et al. [50] argued a variable interaction between *Agrobacterium* strains and plant species.

The difference in growth and branching behavior observed among HR clones might to be due to variations in the presence and expression of *rolA*, *B*, and *C* genes in the individual lines, which could alter the biosynthesis of endogenous growth regulators or the sensitivity of plant cells to growth regulators. The metabolic and growth kinetics differences in HR lines depend on the site of integration of T-DNA into the plant genome [51]. In this work, we confirmed the transformation of the putative HR after the molecular detection of genes *rolC* (which must be present) and *virC1* (a strictly bacterial gene which must be absent); this combination was firstly reported in *Salvia* species in *S. wagneriana* and in other species, as *Linum austriacum*, for instance [49,52].

Not all the selected transformed lines showed good growth performances; the best growth index was obtained with line FA8 which was cultured in a flask and in a bioreactor. In our study, after 42 days of the scale-up process, the hairy root showed a dark brown color in the center, due to viability loss, and clear and vital roots that had begun to turn brown externally. This behavior could be related to the decrease in nutritive compounds in the medium that bring to ageing and/or cell death; this is also observed by Kuzma et al. [53] for the hairy roots of *S. sclarea* after 30 days of culture. Media composition could have a significant impact on hairy root growth in culture systems [54]. In the present study, both MS0 and ½ WPM media achieved the best dry biomass production. Sucrose represents the principal carbon source in plant culture due to its effective absorption through the cell membrane [55]. The determining role of sucrose concentration on the biomass production yield of hairy root culture has been recognized comprehensively [56]. According to Chen et al. [57], initial sucrose concentration had a significant influence on the growth of transformed cell cultures of *S. miltiorrhiza*, showing the fastest growth in medium containing 30 g/L sucrose. This concentration was reported to be the best for *Arnica montana* hairy root growth by Petrova et al. [58] The similar value of sucrose (30 g/L) obtained in this study was found to promote the biomass production of *S. corrugata* hairy roots (Figure S5, Supplementary Materials). This result is consistent with other literature studies. In some cases, the increase in carbon concentration led to reduction in biomass. This reduction can be caused by an excessive osmotic contribution or by toxicity of the carbohydrate [59]. Some authors reported this negative effect of high sucrose concentrations, i.e., Thiruvengadam et al. [60] for hairy roots of bitter melon (*Momordica charantia* L.) and Chen et al. [57] for transformed cell suspension cultures of *S. miltiorrhiza*.

The hairy root biomass growth was monitored by the elaboration of the growth curve of the material grown in batch. A typical sigmoid curve was detected, indicating the period in which the tissue has a logarithmic phase of increment. This is also the period for eventual elicitation to increase the subsequent metabolite production [45].

The growth could be also easily and indirectly monitored in a large scale by measurement of the medium conductivity; the inverse relationship between biomass and conductivity was shown by Taya et al. [61]. The decrease in conductivity could be attributed to the intake of nutrients by the growing hairy roots, leading to a decrease in ion concentration. In our cultures a decrease in conductivity up to the fifth week with a consequent increase in the biomass weight was noted. The increase in conductivity during the last culture week (fifth to sixth) could be caused by a cell lyses due to senescence or death, hence the presence of turbid and light brown aspects of the medium. This behavior was also observed by Urbanska et al. [62] for hairy roots and growth of *Platycodon grandiflorum*.

The type of bioreactor used in this study was the temporary immersion system (TIS) RITA^®^; this container allowed us to obtain considerable fresh and dry biomass. However, the non-homogenous repartition and the formation of clumps complicated the biomass production in long-term culture; similar observations were reported by Georgiev [63]. The design of the RITA^®^ bioreactor, based on the water cyclic movement instead of an impeller continuous rotation, allowed us to minimize the stress of hairy root biomass and avoid callus formation [64] producing a better plant material for extraction (3.5 ± 0.1 g/bioreactor). Nevertheless, the growth rate observed across the hairy root clones tested was quite heterogeneous, and this was evidenced by the number of bioreactors that could be prepared from the second step (glass vessels) and ultimately by the total biomass (both fresh and dry) produced in the bioreactors, although no statistical comparisons could be made due to the lack of replicates.

The research in hairy root production of secondary bioactive metabolites in *Salvia* spp. is still little developed [8]. Apart from many papers in the literature concerning the establishment of hairy root culture of *S. milthiorrhiza* and their elicitation to produce tanshinones [48,65,66,67,68,69,70,71,72,73,74,75,76], only a few other species have been investigated so far. To the best of our knowledge, hairy roots were obtained for *S. austriaca* [77,78,79], *S. broussonetii* [80], *S. bulleyana* [47], *S. castanea* [66,81]*, S. eremophila* [82], *S. macrosiphon* [82], *S. multicaulis* [82], *S. nemorosa* [82], *S officinalis* [46], *S. reuteriana* [82], *S. sclarea* [83,84], *S. tomentosa* [85], *S. verticillata* [82], *S. virgata* [82], *S. viridis* [86], and *S. wagneriana* [49].

Common metabolites of the roots of *Salvia* species are diterpenoids with abietane skeletons [2,9,87,88,89,90,91,92,93,94,95,96,97,98,99,100,101,102,103] that are known to have role in plant chemical defense [104,105] and display a wide spectrum of biological properties [9]. Chemical analysis of the hairy roots of *S. austriaca*, *S. broussonetii*, *S. castanea*, *S. miltiorrhiza*, and *S. sclarea* (Table S4, Supplementary Materials) highlighted the presence of abietane diterpenoids. Compounds with 11,12-*ortho*-quinone abietane skeletons (tanshinone I, tanshinone IIA, tanshinone IIB, dihydrotanshinone I, cryptotanshinone, tetrahydrotanshinone) are characteristic of species belonging to Subg. *Glutinaria* Raf. [106] (*Salvia*s.l. clade IV [107]). Other abietanoids are ascribed to species included in *Salvia* s.s. clade I-C [107], specifically compounds with 7,9(11),13-abietatriene-12-one moiety (taxodone, 6,11-dihydroxyabieta-7,9(11),13-trien-12-one), 7,9(11),13-abietatriene-6,12-dione moiety (taxodione, 7-(2’-oxohexyl)-taxodione), 5(6),7,9(11),13-abietatetraene-12-one moiety (15-deoxy-fuerstione), abieta-8,11,13-trienes (iguestol), 11-hydroxy-12-methoxyabietatriene, ferruginol, 1-oxo-ferruginol, sugiol, cryptojaponol, inuroyleanol, demethylcryptojaponol, pisiferal, 14-deoxycoleon U, 6*R*-hydroxydemethylcryptojaponol, 7-oxodehydroabietane), abietatrien-20-7-lactones (carnosic acid, deoxocarnosol 12-methyl ether), 11,12-orthoquinone diterpenoids characterized by the 4,5-*seco*-abietane skeleton (aethiopinone, 1-oxo-aethiopinone), 11,14-*p*-12-hydroxyquinone diterpenoid (salvipisone), and dimeric abietane diterpenoids (broussonetone A and broussonetone B). Icetexane diterpenoids [25] were described only for *S. broussonetii* (brussonol and demethylsalvicanol). Hairy root cultures have the ability to produce a metabolite pattern similar to untransformed roots [108]. The secondary metabolites produced by the hairy roots can be in higher amounts than in non-transformed ones, and sometimes they are new in comparison to host plants [7] because of disturbances in metabolism caused by the natural transformation or by the insertion of specific genes for specific metabolic pathways [51,109,110]. In the present work, abietane diterpenoids, belonging to the same chemical class of compounds isolated from the aerial parts of *S. corrugata* [33], were found in the roots and hairy roots of this species. The *abeo*-abietane compounds (icetexanes) isolated from the aerial parts [32,33] as well as regenerated shoots and micropropagated plants of *S. corrugata* [34] were not found in the roots nor in the hairy roots. The analysis of the methanolic extract of the hairy roots of *S. corrugata* in comparison to the roots from field-grown plants showed that only one abietane diterpenoid, namely ferruginol (**1**), was contained in both the extracts, while agastol (**5**) was undetected in the untransformed biomass. Our work showed that hairy roots of *S. corrugata* can represent a suitable source of ferruginol [48]. Agastol, also named agastanol [26], was first isolated from *Agastache rugosa* [26,43]. To the best of our knowledge, this is the first report of the presence of this 19(4→3)-*abeo*-abietane diterpenoid in hairy root biomass tissue.

## 4. Materials and Methods

### 4.1. General Experimental Procedures

NMR experiments were performed on a Bruker DRX-600 spectrometer (Bruker BioSpinGmBH, Rheinstetten, Germany) equipped with a Bruker 5 mm TCI CryoProbe at 300 K and a Bruker DRX-400 spectrometer. All 2D NMR spectra were acquired in CDCl_3_ and standard pulse sequences and phase cycling were used for TOCSY, COSY, ROESY, NOESY, HSQC, and HMBC spectra. The NMR data were processed using UXNMR software. The ROESY spectra were acquired with *t*_mix_ = 400 ms. HRESIMS spectra were acquired in the positive ion mode by an LTQ Orbitrap XL mass spectrometer (Thermo Fisher Scientific, San Jose, CA, USA). The Orbitrap mass analyzer was calibrated according to the manufacturer’s directions by using a mixture of caffeine, methionine-arginine-phenylalanine-alanine-acetate (MRFA), sodium dodecyl sulfate, sodium taurocholate and Ultramark 1621. Data were collected and analyzed using the software provided by the manufacturer. LC/MS/MS analyses were performed on a Q-Trap 6500 spectrometer (ABI SCIEX, Milano, Italy) coupled with a Nexera UHPLC system (Shimadzu Italia, Milano, Italy). MPLC chromatography was performed on a Spot Liquid Chromatography system (Armen Instrument, Saint Ave, France) with Normal Phase Si60 Cartridges Supervarioflash and LiChroprep RP-18 (40–63 µm) (Merck, Darmstadt, Germany). Silica gel 60 F254-coated aluminum sheets (Merck, 20 × 20 cm, 0.2 mm layer thickness, Darmstadt, Germany) were used for TLC. CHCl_3_-CH_3_OH-HCOOH (10:0.5:0.1) was used as the mobile phase, and spots were detected by spraying 50% H_2_SO_4_, followed by heating. Semi-preparative HPLC was carried out using a Waters W600 pump equipped with a Rheodyne Delta 600 injector, a 2414 refractive index detector, and a 2998 photodiode array detector (all Waters Corporation, Milford, MA, USA). A Symmetry C18 Prep Column, 7.8 × 300 mm ID, with a 7 µm particle size (Waters Corporation, Milford, MA, USA) was used at room temperature, with a flow rate of 2.0 mL/min, sample loop 100 µL, eluents A: H_2_O, B: CH_3_OH, gradient: 5% to 100% B in 61 min, 100% B to 75 min.

### 4.2. Plant Material

Fresh aerial parts and roots of *S. corrugata* were obtained from plants grown in field and open air at the Agricultural Research Council Unit CREA-OF located in Sanremo (Italy). The species was identified by Dr. Gemma Bramley and a voucher specimen is deposited at Kew Herbarium (K) (Kew, Surrey, UK).

### 4.3. Establishment of Hairy Root Cultures

Apical and nodal explants of *S. corrugata* were sterilized as follows: washing with tap water for 15 min followed by treatment with 1% of active chlorine supplemented with few drops of Tween 20 for 15 min. The explants were rinsed three times with sterile distilled water for 10 min each. The sterile shoots were grown onto solid MS Murashige and Skoog [111] base medium, 30 g/L of sucrose, and 8 g/L plant agar. The medium pH was adjusted using KOH and HCl to 5.7 before autoclaving at 1 atm and 121 °C for 20 min. The shoots were grown at 23 ± 2 °C under a day-night illumination regime (photoperiod 16:8) by a white fluorescent Philips Master TL-D 36 W/840 lamp flux density 35 µE.m^−2^·s^−1^ and the medium was renewed monthly. To obtain shoot multiplication for maintaining the genotype in vitro, 0.3 mg/L of Benzyl adenine (BA) was added to the base medium [112]. The transformation was performed using two strains of *A. rhizogenes*: *A. rhizogenes* wild-type ATCC 15834 (glycerol stock from CREA-OF Sanremo (IM), Italy) and the hypervirulent *A. rhizogenes* strain LBA 9402 (a gift from Prof. Laura Pistelli, University of Pisa, Italy), selected on the basis of literature data [46,48,49,113]. The bacteria were grown, respectively, onto semi-solid medium (1.5% agar) NB and YMB [114] in the dark. For each strain, a single bacterial colony was selected and transferred into a 50 mL sterile polypropylene conical tube containing 10 mL of the same medium without agar for overnight growth at 28 °C and under agitation at 120 rpm. Optical density was measured by a spectrophotometer (O.D.600 nm = 0.3 for ATCC 15834 and 0.35 for LBA 9402). The leaves of the second and third node of the shoots of *S. corrugata*, grown in MS hormone-free medium for 4 weeks, were isolated, gently wounded on both sides of the leaf tissue and on the central rib, immersed into 5 mL of the previous bacterial suspension and swirled for 20 min at 24 °C. The control of the experiment was carried out by treating explants with sterile distilled water for the same time and at the same temperature. All sub-cultured explants were laid horizontally on sterile Petri plates with solid hormone-free MS medium and incubated at 24 °C in dark for 3 days. The explants were then transferred every 15 days onto MS medium containing cefotaxime (100 mg/L of cefotaxime was added for the strain ATCC 15834, while 500 mg/L was used for strain LBA 9402). The transformation efficiency after 30 days from the co-culture was determined as the percentage of the number of explants that developed putative hairy root to the total number of explants used for transformation. Single roots were excised from the leaf explants and transferred to single Petri dish with agarized MS medium containing the amount of cefotaxime for each bacterial strain, 100 mg/L for ATCC 15934 and 500 mg/L for LBA 9402. Among the different roots developed, only those with specific phenotypic characteristics of hairy roots (i.e., rapid branching, missing geotropism, fast growth rate and development of white hairs after the growth on hormone-free medium) were considered as putative HR material, selected, and maintained for further studies. After 30 days, the selected clones of roots derived from ATCC 15834 and LBA 9402, based on length and number of branchings were transferred on fresh MS medium containing cefotaxime concentration reduced, respectively, to 50 mg/L and 300 mg/L for 30 additional days; in the further transfer of the material, the cefotaxime was eliminated. Root clones were sub-cultured onto the same solid medium every month. After two months of sub culturing without antibiotic, the axenic hairy roots were transferred to MS0 liquid medium and tested for liquid adaptability.

The root viability was performed using calcein acetoxymethylester (AM) and fluorescein diacetate (FDA). An aliquot of the calcein AM stock solution 33.33 mg/mL (53.54 mM) was diluted 50 times with distilled water to obtain a solution of about 1 mM. The solution of FDA stock solution was prepared by diluting FDA in acetone (5 mg/mL) and stored at −18 °C. Immediately before staining, a sample of this solution was diluted 100 times with distilled water to make the final solution (50 µg/mL) and laid over the fresh material. Living hairy roots were immersed in a drop of these solutions and kept for 15 min (FDA) and 30 min (calcein AM) at room temperature and in dark conditions. The material was mounted on the microscopic glass slides and observed with the bright field and fluorescence microscopy (LEICA DM 4000 B with GFB filter cube: excitation range blue, excitation filter BP 470/40, dichromatic mirror 500, suppression filter BP525/50) and the pictures were taken with LEICA DFC 350 FX. To detect the presence of terpene inside the diverse structure of plant tissues, the Nadi protocol was used [115,116]. Nadi specifically stains lipids in blue and terpenes in purple. The dye was prepared using α-naphthol (0.5 mL, 1%, *v*/*v* in 40% ethanol) and dimethylphenylenediamine-HCl (0.5 mL, 1%, *v*/*v*) in sodium phosphate buffer pH 7 (5 mL, 50 mM). A few minutes after immersion in the tube containing the dye, the fragment of hairy root was picked up, mounted on glass slides, and observed under the microscope.

### 4.4. Confirmation of Transformation

Detection of Ri T-DNA integration. The genomic DNA of selected clones of putative hairy roots and of a negative control from non-transformed roots (normal root from in vitro plant) was extracted from 100 mg of plant tissue by Dneasy Plant Mini Kit (Qiagen^®^). The DNA concentration was quantified using a NanoDrop^®^ 2000 Spectrophotometer (Thermo Scientific, Waltham, MA, USA) [50,117]. The positive control was represented by plasmid DNA of *Agrobacterium rhizogenes* strain ATCC 15834 carried out according to the Klimyuk method [118]. DNA samples were used as a template for PCR to determine the presence of the *rolC* gene in the T-DNA (i.e., root transformation) and *virC1* gene (i.e., presence/absence of bacterial contamination). The PCR was achieved in a BIO RAD T100TM Thermal cycler (Bio Rad, Hercules, CA, USA) and was used to detect the Ri T-DNA integration in the plant genome. The analysis was performed to target two specific genes of *A. rhizogenes*, namely *rolC* and *virC1*. The eventual amplification of the *virC1* gene fragment indicates the presence of the contamination of *A. rhizogenes* bacterial cells or the residual presence of bacterium in the plant tissues; this was detected by amplification of a 326 bp using *virC1*-specific primers (PVIRC2775 5′-CTCGCTCAGCAGCAGTTCAATG-3′ and PVIRC3101 5′-GACGGCAAACGATTGGCTCTC-3′) [119]. The presence of the *rolC* gene was checked by PCR amplification of a 514 bp fragment (forward Primer (5′-CGACCTGTGTTCTCTCTTTTTCAAGC-3′) and reverse primer (5′-GCACTCGCCATGCCTCACCAACTCACC-3′)). Each reaction for the amplification of the 514 bp fragment of the *rolC* gene, and 326 bp fragment of the *virC1* genes was performed with plant DNA diluted 1:2 with distilled water and specific primer. The PCR reaction was performed in a final volume of 25 µL with 1 µL of DNA solution, 3 µL of PCR Rxn buffer (-MgCl_2_), 0.9 µL of MgCl_2_, 0.6 µL of dNTPs, 0.5 µL of each primer, 0.3 µL of platinum^®^ Taq (DNA polymerase) and 18.2 µL of water. The *rolC* PCR condition gene followed Scorzas’s cycling program [120], modified as follows: initial denaturation at 94 °C for 1 min, 35 cycles at 94 °C for 30 min denaturation, 65 °C for 30 min annealing, 72 °C for 1 min elongation, and a final elongation step of 5 min at 72 °C. The absence of bacterial contamination of the plant tissue was confirmed after PCR amplification of 326 bp fragment of the *virC1* gene using the same PCR program. Amplification products of these genes were detected after electrophoresis analysis on agarose gel 1.5% in TAE buffer and stained with ethidium bromide.

### 4.5. Growth of Hairy Root Cultures

The experiments of plant material growth were performed on the lines confirmed with morphological and molecular evidence as hairy root.

#### 4.5.1. Effect of Medium Composition

To optimize the suitable culture medium for the growth of hairy roots, three common culture media formulations were tested, namely MS, B5 [121], and WPM [122] at full concentration or at half strength: ½ MS, ½ B5, and ½ WPM. Hairy roots were cultivated in 0.5 L TIS bioreactor RITA^®^ containing 150 mL of the hormone-free liquid basal medium supplemented with 30 g/L sucrose. Cultivation was performed with an immersion frequency of 3 min flooding every 3 h of stand-by periods in the dark at 23 ± 2 °C for 30 days. A total of 3 g of fresh weight biomass was used for each bioreactor. For each treatment, four independent biological replicates were used. At the end of the culture period of 30 days, the growth rate of each culture was determined (FW, DW).

#### 4.5.2. Effect of Initial Sucrose Concentration

A total of 3 g of hairy roots were used as inoculums in 0.5 L TIS bioreactor RITA^®^ containing 150 mL of MS hormone-free liquid medium supplemented with 2%, 3%, and 4% (i.e., 20, 30, 40 g/L) sucrose, respectively, with an immersion frequency of 3 min flooding every 3 h of stand-by periods in the dark at 23 ± 2 °C, as already described above. For each treatment, four independent biological replicates were used. After 30 days of culture, the biomass produced was harvested and analyzed.

#### 4.5.3. Growth Kinetics

The growth curve of fresh and dry weight was carried out on clone FA8 for 42 days. The experiment was performed in a 250 mL glass vessel with a transparent cap. Twenty-one replications were prepared at *t*_0_, each by inoculating 1 g of fresh hairy root accurately weighed into 50 mL of MS hormone-free medium, incubated at dark conditions under rotation at 120 rpm. At intervals of 7 days, three samples were randomly chosen and analyzed for fresh and dry weight (FW, DW) evaluation, pH, and conductivity. Fresh hairy roots were filtered, blotted with tissue paper, and the fresh root weight was measured. For dry weight measurement, the fresh hairy roots were dried in an oven at 60 °C for 12 h and then weighed. The experiment was made in batch culture without changing or filling the medium.

#### 4.5.4. Scale up Production

The three-step culture sequence (Petri dish, glass vessel, 0.5 L bioreactor) was applied for biomass production. Growth units in bioreactors containing 150 mL of the MS0 medium were prepared with 5 g aliquots of material produced in the glass vessels step. A total number of 13, 4, and 1 bioreactors for the FA13, FA8, and FL7 clones, respectively, could be produced. The liquid medium MS0 was completely renewed every 10 to 15 days with a fresh one. Three months after culture into the bioreactors, the total biomass in bioreactor was harvested and stored at −80 °C before lyophilization.

### 4.6. Phytochemical Analysis

#### 4.6.1. Extraction of the Plant Material

The dried untransformed roots (219.3 g) and hairy roots (clone SCO-HR-FA8, 46.0 g) of *S. corrugata* were exhaustively extracted with methanol, affording 17.4 g and 16.9 g methanolic extract, respectively.

#### 4.6.2. Determination of the Content of Demethylfruticuline A and Fruticuline A in the In Vitro Biomass

LC/MS/MS analyses were carried out to reveal and eventually quantify fruticuline A and demethylfruticuline A in the different extracts.

Pure compounds were used to set up and validate the method. Mass spectra were acquired in positive multiple reaction monitoring (MRM) mode, to maximize selectivity and sensitivity. The transitions 325.3–241.0 and 311.2–227.0 were selected for fruticuline A and demethylfruticuline A, respectively. Chromatography was performed on a Kinetex C18 column (50 × 2.1 mm, 1.7 µm, Phenomenex, Torrance, CA, USA) using a mixture of 0.1% formic acid in water (Eluent A) and 0.1% formic acid in acetonitrile (Eluent B) as the mobile phase. Compound elution was achieved through a gradient from 30% to 70% of B over 5 min. Using this method, a Lower Limit of Detection (LLOD) of 0.1 µg/mL was measured for both compounds, whereas the Lower Limit of Quantization (LLOQ) was 0.3 µg/mL, and the response was linear over a 0.3–30 µg/mL concentration range (Figure S8, Supplementary Materials). Different samples were analyzed in triplicate, injecting 3 µL of each 1 mg/mL extract.

#### 4.6.3. Analysis of the Extracts

The methanolic extract of the roots was fractionated by Si gel MPLC eluting with n-hexane/CHCl_3_/CH_3_OH at concentrations varying from 100:0:0 to 0:0:100 to obtain 12 fractions (I–XII). Fraction III (59.8 mg) (eluted with CHCl_3_, from 0.66 L to 0.75 L) was purified by semi-preparative RP HPLC to obtain **1** (2.5 mg). Fraction VI (280.0 mg) (eluted with CHCl_3_, from 0.75 L to 0.84 L) was purified by semi-preparative RP HPLC to obtain **2** (12.6 mg) and **3** (17.5 mg). Fraction V (1.5 g) (eluted with CHCl_3_, from 0.84 L to 1.02 L) was further purified by Si gel MPLC eluting with n-hexane/CHCl_3_/CH_3_OH at concentrations varying from 100:0:0 to 0:0:100 to obtain 29 fractions (I_i_–XXIX_i_). Fraction X_i_ (166.6 mg) (eluted with CHCl_3_, from 0.84 L to 0.93 L) was purified by semi-preparative RP HPLC to obtain 2 (15.0 mg) and **4** (13.6 mg). Fraction XI_i_ (70.7 mg) (eluted with CHCl_3_/CH_3_OH 95:5, from 1.32 L to 1.41 L) was purified by semi-preparative RP HPLC to obtain 2 (4.4 mg) and 4 (3.3 mg). Fraction VI (1.5 g) (eluted with CHCl_3_/CH_3_OH 95:5, from 1.02 L to 1.20 L) was purified by semi-preparative RP HPLC to obtain a mixture of ursolic and oleanolic acid (94.0 mg).

The methanolic extract of the hairy roots was fractionated by Si gel MPLC eluting with *n*-hexane/CHCl_3_/CH_3_OH at concentrations varying from 100:0:0 to 0:0:100 to obtain 14 fractions (I*_ii_*–XIV*_ii_*). Fraction III_ii_ (107.6 mg) (eluted with CHCl_3_, from 0.848 L to 0.54 L) was purified by semi-preparative RP HPLC to obtain **1** (4.5 mg) and **5** (7.7 mg).

#### 4.6.4. Determination of the Content of Agastol and Ferruginol

Quantification of ferruginol and agastol in the extract of the hairy roots was carried out following the reverse-phase HPLC analytical method of Okamura et al. [123] with slight modifications. The mobile phase was a mixture of water–methanol containing 0.1% formic acid. Gradient separation started from 25% methanol all the way to 100% in 30 min, and stayed at 100% for another 20 min. The flow rate was 0.4 mL/min. The column temperature was kept at 25 °C and the injection volume was of 20 μL. The acquisition wavelength used for both the compounds was 220 nm. The analysis time was 50 min. The retention times of agastol and ferruginol were 40.45 and 41.19 min, respectively. No interfering peaks were detected at the retention times of the analytes. Calibration curves were constructed over the concentration range (0.1–1.0 mg/mL). Standard solutions of agastol and ferruginol in methanol were prepared at five different concentrations (0.1, 0.2, 0.3, 0.5, 1.0 mg/mL). Calibration curves were linear (R^2^ values were 0.9977 and 0.9983, respectively) and detection and quantitation limits (computed according to the equations LOD = 3.0δ/b, LOQ = 10.0δ/b) were adequate for the purposes of the study (agastol: LOD = 0.0010 mg, LOQ = 0.0085 mg; ferruginol: LOD = 0.0012 mg, LOQ = 0.0098 mg). Quantitative results, expressed as substance relative amount (%, *w*/*w*) in the methanolic extract, are reported as 95% confidence intervals.

### 4.7. Statistical Analysis

Statistical analysis was performed using a one-way ANOVA test. All the experiments were performed in triplicate. The data are presented in the tables and in the figures as means ± SD. Different symbols show significantly different values. Mean values were considered significantly different at *p* < 0.05.

## 5. Conclusions

In conclusion, this is the first study on the establishment of hairy root cultures of *S. corrugata*. The results indicate that the types of *A. rhizogenes* and the election of hairy root clones represent an important factor to have a huge final biomass accumulation. Additionally, the biomass production is influenced both by the medium composition in terms of formulation and the sucrose amount. The modulation of conductivity during the growth period could give information of the state of culture. The qualitative composition of their methanol extracts in comparison with untransformed roots was evaluated. Ferruginol (**1**) was contained in both extracts, while agastol (**5**) was produced only by hairy root culture. The quantitative study of the content of these diterpenes showed that the hairy roots of *S. corrugata* can be considered a source of these bioactive molecules. Further studies will be conducted to evaluate whether elicitation can increase the production of bioactive compounds [7], as the use of elicitors is one of the most common strategies to induce the accumulation of a bioactive secondary metabolites [124,125].

## Figures and Tables

**Table 1:** Induction of roots by different strains of *A. rhizogenes* vs. non treated control, 30 days after inoculation ^a^.

*A. rhizogenes* Strain	N° of Treated Explants	N° of Explants Producing Roots and Relative Percentage
ATCC 15834	16	12 (75%)
LBA 9402	21	8 (38.1%)
Control	21	3 (14.3%)

^a^ Only viable fragments that give rise to root neo-organogenesis are reported.

**Table 2:** Biomass production in bioreactor ^a^.

Clones	Number of Bioreactors (Total Liquid Volume)	FW (g)/Bioreactor (FW/L of Total MS Used)	DW (g)/Bioreactor) (DW/L of Total MS Used)	Total FW Produced (g)	Total DW Produced (g)
SCO-HR-FA8	13 (15.6 L)	37.5 ± 2.3 ^a^ (31.23 ± 1.9)	3.5 ± 0.1 ^b^ (2.9 ± 0.1)	487.8	46.0
SCO-HR-FA13	4 (4.8 L)	35.4 ± 2.4 ^a^ (29.5 ± 2)	4.16 ± 0.38 ^c^ (3.5 ± 0.3)	143.4	16.5
SCO-HR-FL7	1 (1.2 L)	23.3	2.4	23.3	2.4

^a^ Values are presented as means ± standard deviation. Different letters indicate significant differences between groups at *p* ≤ 0.05).

## Data Availability

The data presented in this study are available upon request from the corresponding author.

## Supplementary Materials

Figure S1: Hairy root induction, Figure S2: Viability of hairy roots, Figure S3: Terpene content of hairy roots, Figure S4: Effect of different medium formulations on hairy root growth in bioreactor, Figure S5: Effect of sucrose concentration on hairy root growth, Figure S6: Growth curve of *S. corrugata* hairy roots, Figure S7: Medium conductivity, Table S1: Root selection after 30 days of induction from leaves explants of *S. corrugata*, Table S2: Daily increases of length of the principal root and branching index (number of branches in 1 month/30 days) of different clones from wild type ATCC 15834, Table S3: Daily increases of length of the principal root and branching index (number of branches in 1 month/30 days) of different clone strain LBA 9402. Table S4: Literature survey on *Salvia* species studied for hairy root establishment and production of secondary metabolites.

## Sample Availability

Samples of the compounds are available from the authors.
