# Supplementary material for: Abietane Diterpenoids from the Hairy Roots of Salvia corrugata
Source: Molecules. 2021 Aug 25;26(17):5144. doi: 10.3390/molecules26175144 (PMC8434070; doi:10.3390/molecules26175144)
Supplement: Supplementary file 1 [file molecules-26-05144-s001.zip › molecules-1310857-supplementary.pdf]

Supplementary Material for:

Abietane diterpenes from hairy roots of Salvia corrugata

Roméo Arago Dougué Kentsop [1,2], Valeria Iobbi [1], Giuliana Donadio [3], Barbara Ruffoni [2], Nunziatina De Tommasi [3*] and Angela Bisio [1,*]

[1] Dipartimento di Farmacia, Università di Genova, Via Brigata Salerno 13, 16147 Genova, Italy; bisio@difar.unige.it; dougue.kentsop.phd@difar.unige.it; valeria.iobbi@edu.unige.it

[2] Consiglio per la Ricerca e la Sperimentazione in Agricoltura – CREA Centro di ricerca Orticoltura e Florovivaismo, San Remo (IM), Italy; barbara.ruffoni@crea.gov.it

[3] Dipartimento di Farmacia, Università di Salerno, Via Giovanni Paolo II 132, 84084 Salerno, Italy; gdonadio@unisa.it , detommasi@unisa.it

* Correspondence: bisio@difar.unige.it; detommasi@unisa.it;Tel.: (A. B: +39 010 3352637; N. DeT. +39 089 969754)

CONTENT

Figure S1. Hairy root induction.
Figure S2. Viability of hairy roots.
Figure S3. Terpene content of hairy roots.
Figure S4. Effect of different medium formulations on hairy root growth in bioreactor
Figure S5. Effect of sucrose concentration on hairy root growth.
Figure S6. Growth curve of *S. corrugata* hairy roots.
Figure S7. Medium conductivity.
Figure S8. Calibration curves obtained for the LC/MS/MS analysis of fruticuline A and demethylfruticuline A using pure compounds.

Table S1. Root selection after 30 days of induction from leaves explants of *S. corrugata*. 6
Table S2. Daily increases of length of the principal root and branching index (number of branches in 1 month/30) of different clones from wild type ATCC 15834 6
Table S3. Daily increases of length of the principal root and branching index (number of branches in 1 month/30) of different clones strain LBA 9402. 7
Table S4. Literature survey on *Salvia* species studied for hairy root establishment and production of secondary metabolites...8

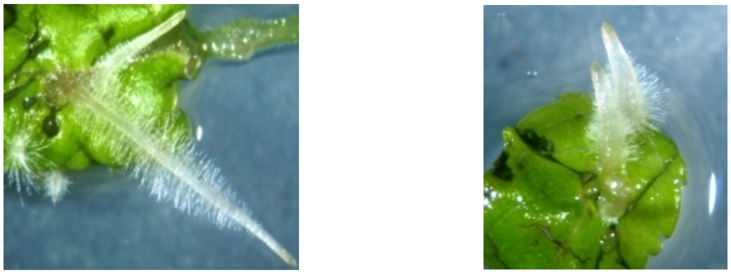

(a) **(b)**

Figure S1. Hairy root induction.
Hairy roots of *S. corrugata* induced at the wounded sites of the explants 14 days after treatment by *A. rhizogenes* strain: (a) ATCC 15834 and (b) LBA 9402.

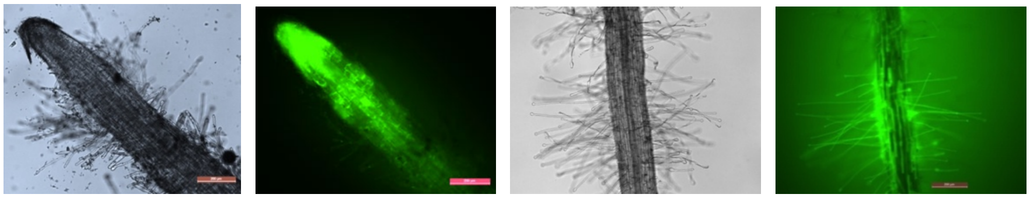

(a) **(b)** **(c)** **(d)**

Figure S2. Viability of hairy roots.
Bright field and fluorescence micrographs of stained hairy roots: (**a)** and (**b**): Calcein AM; (**c**) and (**d**): FDA. (10×). Bar = 200 µm

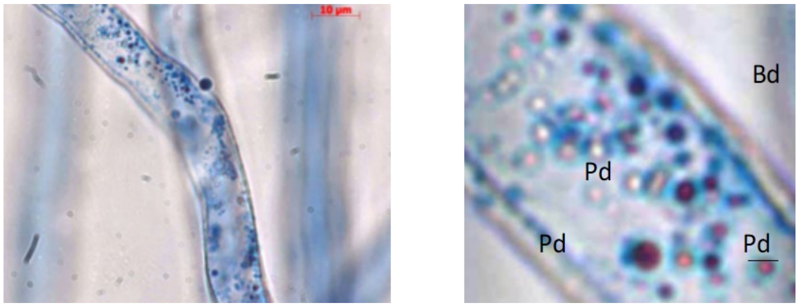

(a) **(b)**

Figure S3. Terpene content of hairy roots.
Bright field micrographs of hairy roots of *S. corrugata* stained with Nadi reagent, showing the presence of terpenes (purple) and lipids (blue) droplets. (60×). Bars = 10 µm

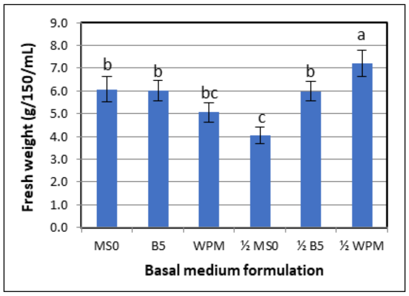

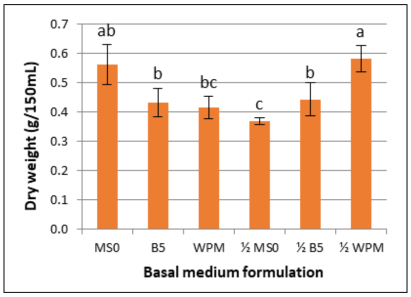

(**a**) (**b**)

Figure S4. Effect of different medium formulations on hairy root growth in bioreactor
(**a**) Fresh weight; (**b**) Dry weight. Values represent the mean ± standard deviation (SD), n = 4; different letters identify values which differ at $p \leq 0.05$.

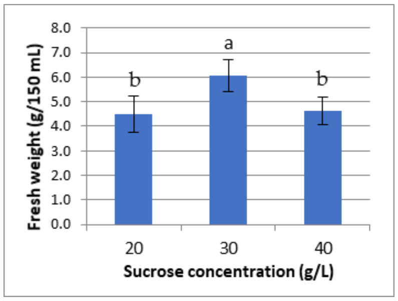

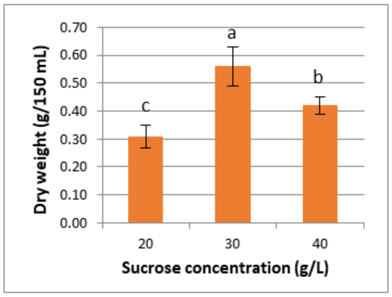

(**a**) (**b**)

Figure S5. Effect of sucrose concentration on hairy root growth.
(**a**) Fresh weight; (**b**) Dry weight. Values represent the mean ± standard deviation (SD), n = 4; different letters identify values which differ at $p \leq 0.05$.

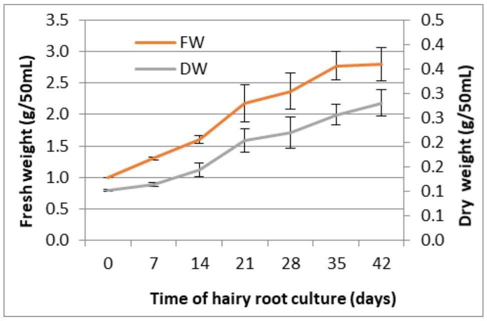

Figure S6. Growth curve of *S. corrugata* hairy roots.

Culture of clone FA8 in MS0 liquid medium in the dark at 120 rpm. FW (fresh weight) and DW (dry weight); 1 g of fresh hairy root was cultured in 250 mL glass vessels with 50 mL of MS0 liquid medium for 6 weeks and sampled at an interval of one week.

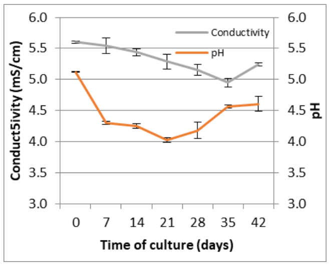

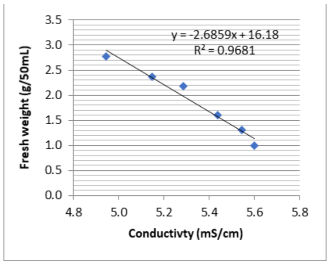

(**a**) (**b**)

Figure S7. Medium conductivity.

(**a**) Evolution of medium conductivity and pH at different stages of growth. Determinations were recorded every 7 days. (**b**) Decrease of medium conductivity in relation to the increase of hairy root fresh weight during the 5 first weeks of culture.

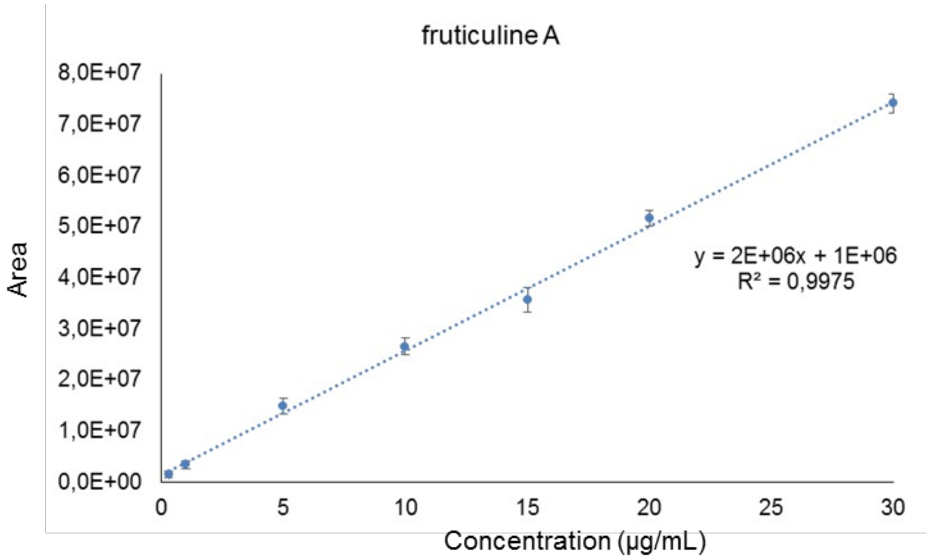

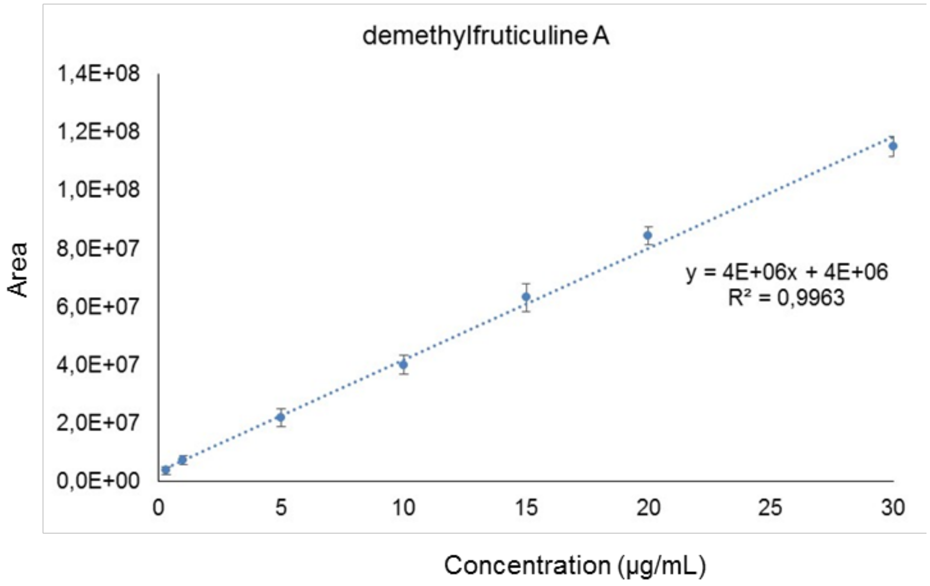

Figure S8. Calibration curves obtained for the LC/MS/MS analysis of fruticuline A and demethylfruticuline A using pure compounds.

Table S1. Root selection after 30 days of induction from leaves explants of *S. corrugata*.

A. rhizogenes strain	Total number of roots	Mean number of roots per explants	Number of roots excised and transferred
ATCC 15834	16	2.7	26
LBA 9402	21	2.6	8
Control (-)	21	1.7	1

Table S2. Daily increases of length of the principal root and branching index (number of branches in 1 month/30) of different clones from wild type ATCC 15834

A. rhizogenes ATCC 15834	Increase (30 days later)		Daily increase		Missing of geotropism
**clones**	**branch number**	**length (mm)**	**branching index**	**length (mm)**	
SCO-HR-FA1	2	20	0.07	0.67	++
SCO-HR-FA2	1	30	0.03	1.00	+++
SCO-HR-FA3 [1]	0	0	0.00	0.00	-
SCO-HR-FA4 [1]	0	0	0.00	0.00	-
SCO-HR-FA5	1	30	0.03	1.00	-
SCO-HR-FA6 [1]	0	0	0.00	0.00	-
SCO-HR-FA7 [1]	0	0	0.00	0.00	-
SCO-HR-FA8 [2]	6	30	0.20	1.00	+++
SCO-HR-FA9	1	1	0.03	0.03	-
SCO-HR-FA10 [1]	0	0	0.00	0.00	-
SCO-HR-FA11	0	0	0.00	0.00	-
SCO-HR-FA12	0	10	0.00	0.33	+
SCO-HR-FA13 [2]	4	30	0.13	1.00	+++
SCO-HR-FA14 [1]	0	0	0.00	0.00	+
SCO-HR-FA15	0	15	0.00	0.50	+
SCO-HR-FA16	0	2	0.00	0.07	-
SCO-HR-FA17 [1]	0	0	0.00	0.00	-
SCO-HR-FA18 [1]	0	0	0.00	0.00	-
SCO-HR-FA19	3	29	0.10	0.97	++
SCO-HR-FA20 [1]	0	0	0.00	0.00	-
SCO-HR-FA21	2	16	0.07	0.53	+
SCO-HR-FA22	0	1	0.00	0.03	-
SCO-HR-FA23 [1]	0	0	0.00	0.00	-
SCO-HR-FA24	1	1	0.03	0.03	-
SCO-HR-FA25	0	12	0.00	0.40	+
SCO-HR-FA26	1	18	0.03	0.60	+

[1] Died clones after 1 month.

[2] Selected clones.

Table S3. Daily increases of length of the principal root and branching index (number of branches in 1 month/30) of different clones strain LBA 9402.

A. rhizogenes ATCC 15834	**Increase (30 days later)**		**Daily increase**		**Missing of geotropism**
**clones**	**branch number**	**length (mm)**	**branching index**	**length (mm)**	
SCO-HR-FL1	0	3	0.00	0.10	-
SCO-HR-FL2	1	28	0.03	0.93	+
SCO-HR-FL3	0	1	0.00	0.03	-
SCO-HR-FL4	8	21	0.27	0.70	+
SCO-HR-FL5	0	10	0.00	0.33	+
SCO-HR-FL-6	1	24	0.03	0.80	+++
SCO-HR-FL7 [2]	10	21	0.33	0.70	+++
SCO-HR-FL-8 [1]	0	0	0.00	0.00	-

[1] Died clones after 1 month.

[2] Selected clones.

Table S4. Literature survey on *Salvia* species studied for hairy root establishment and production of secondary metabolites.

**Accepted name - Protologue** [1]	Synonyms [1]	Molecular phylogenetic studies	Lifeform [1]	Geographical distribution	Compounds detected in the hairy roots [References]
Salvia austriaca Jacq**.** **-** Fl. Austriac. 2: 8 (1774)	Homotypic Names: *Elelis austriaca* (Jacq.) Raf., Fl. Tellur. 3: 93 (1837); *Sclarea austriaca* (Jacq.) Soják, Cas. Nár. Mus., Odd. Prír. 152: 21 (1983). Heterotypic Names: *Salvia sclaraea* Crantz, Stirp. Austr.Fasc., ed.2, 2: 236 (1769), nom. illeg.; *Salvia bavarica* Schrank, Baier. Fl. 2: 133 (1789); *Sclarea distans* Moench, Methodus: 375 (1794); *Salvia distans* (Moench) Pohl, Tent. Fl. Bohem. 1: 29 (1809).	*Salvia* clade I [2], [3]; *Salvia* s.s. clade I-C [4], [5]	Hemicr.	From S. Czechoslovakia & N. Ukraine southwards to NE. Bulgaria	taxodone, taxodione, 15-deoxy-fuerstione, 7-(2'-oxohexyl)-taxodione [6-8]
Salvia broussonetii Benth. - Labiat. Gen. Spec.: 227 (1833)	Heterotypic Synonyms: *Salvia bolleana* de Noé ex Bolle, Bonplandia (Hannover) 8: 284 (1860); *Salvia broussonetii* Bolle, Bonplandia (Hannover) 8: 284 (1860), nom. illeg.	*Salvia* s.s. clade I-C [5]	Cham. or nanophan.	Canary Is. (Tenerife, Lanzarote?)	brussonol, iguestol (6R,11-dihydroxy-12-methoxy-abieta-8,11,13-triene), 7-oxodehydroabietane, 11-hydroxy-12-methoxyabietatriene, taxodione, inuroyleanol, ferruginol, deoxocarnosol 12-methyl ether, cryptojaponol, pisiferal, sugiol, isomanool, 14-deoxycoleon U, 6*R*-hydroxydemethylcryptojaponol, demethylsalvicanol, demethylcryptojaponol [9], broussonetone A, broussonetone B [10]
Salvia bulleyana Diels - Notes Roy. Bot. Gard. Edinburgh 5: 233 (1912)	**-**	*Salvia* s.l. clade IV-A (*Glutinaria* Raf.) [5]; Subg. *Glutinaria* subclade *Eurysphace* (G6) [11]	Hemicr.	China (Yunnan)	rosmarinic acid, rosmarinic acid hexoside, methyl rosmarinate, caffeic acid, caffeoyl-4'-hydro-xyphenyllactic acid, salvianolic acids K, E and F [12]
Salvia castanea Diels - Notes Roy. Bot. Gard.	Heterotypic Names: *Salvia castanea* f. *glabrescens* E.Peter, Acta Horti Gothob. 9: 134 (1934); *Salvia castanea* f. *pubescens* E.Peter, Acta Horti Gothob. 9: 134 (1934); *Salvia castanea* f.	Chinese Clade, subclade iii [13];	Hemicr.	C. Himalaya to SC. China	Cryptotanshinone, tanshinone I, tanshinone IIA, dihydrotanshinone [14,15]

Edinburgh 5: 233 (1912)	*tomentosa* E.Peter, Repert. Spec. Nov. Regni Veg. 39: 181 (1936).	*Salvia* s.l. clade IV-A (*Glutinaria* Raf.) [4], [5];<br><br>Subg. *Glutinaria* subclade *Eurysphace* (G6) [11]		(Sichuan, Yunnan, Xizang)	caffeic acid, salvianolic acid, rosmarinic acid [15]
Salvia corrugata Vahl - Enum. Pl. Obs. 1: 252 (1804)	*Sphacele gaudichaudii* Briq. (in: Annuaire du Conservatoire et Jardin Botaniques de Genève 2: 182. 1898); *Alguelagum gaudichaudii* Briq. (nomen, in: Annuaire Conserv. Jard. Bot. Geneve 2: 182. 1898).	*Salvia* clade II [2], [3];<br><br>"*Uliginosae* clade" Sect. *Corrugatae* [16], [17]	Nanophan.	S. Colombia to Peru	agastol, ferruginol, ursolic acid, oleanolic acid
Salvia eremophila Boiss. - Diagn. Pl. Orient. 5: 12 (1844).	Homotypic Names:<br><br>*Pleudia eremophila* (Boiss.) M.Will, N.Schmalz & Class.-Bockh., Turkish J. Bot. 39: 703 (2015).	Salvia clade III [3]<br><br>Cluster I [18]	Cham.	C. & S. Iran	[19]
Salvia macrosiphon Boiss. - Diagn. Pl. Orient. 5: 11 (1844)	Heterotypic Synonyms: *Salvia kotschyi* Boiss., Diagn. Pl. Orient. 7: 46 (1846); *Salvia macrosiphon* var. *cabulica* Benth. in A.P.de Candolle, Prodr. 12: 282 (1848); *Salvia macrosiphon* var. *kotschyi* (Boiss.) Boiss., Fl. Orient. 4: 615 (1879); *Salvia macrosiphonia* St.-Lag., Ann. Soc. Bot. Lyon 7: 134 (1880); *Salvia cuspidatissima* Pau, Trab. Mus. Ci. Nat., Ser. Bot. 14: 33 (1918); *Salvia albifrons* Nábelek, Spisy Prír. Fak. Masarykovy Univ. 70: 49 (1926); *Salvia macrosiphon* var. *brachycalycina* Bornm., Bot. Jahrb. Syst. 62: 238 (1934); *Salvia macrosiphon* var. *glandulosissima* Bornm., Bot. Jahrb. Syst. 62: 238 (1934); *Salvia nachiczevanica* Pobed. in V.L.Komarov, Fl. URSS 21: 657 (1954).	*Salvia* s.s. clade I-C [5]	Hemicr.	SE. Turkey to C. Asia and Arabian Pen.	[19]
Salvia miltiorrhiza Bunge - Enum. Pl. China Bor.: 50 (1833)	-	*Salvia* clade III [2], [3];	Hemicr.	China (Anhui, Hebei, Henan, Hubei, Hunan, Jiangsu, Shaanxi, Shandong,	tanshinone I, tanshinone IIA, tanshinone IIB, dihydrotanshinone I, cryptotanshinone, tetrahydrotanshinone. [21-32];

		Clade III + Chinese and Japanese *Salvia* [20];<br><br>Chinese clade, subclade i, group i [13];<br><br>*Salvia* s.l. clade IV-B (*Glutinaria* Raf.) [4], [5];<br><br>Subg. *Glutinaria* subclade *Drymosphace* (G7) (*S. miltiorrhiza* group) [11]		Shanxi, Zhejiang) [Japan]	(*R*)-2-hydroxy-3-(3,4-dihydroxyphenyl) propionic acid (danshensu), caffeic acid, cinnamic acid, ferulic acid, protocatechuic aldehyde, rosemarinic acid, salvianolic acid B, lithospermic acid B [21-23,25-29,31-35]
Salvia multicaulis Vahl - Enum. Pl. Obs. 1: 225 (1804).	Homotypic Names: *Arischrada multicaulis* (Vahl) Pobed., Novosti Sist. Vyssh. Rast. 9: 247 (1972); *Stiefia multicaulis* (Vahl) Soják, Cas. Nár. Mus., Odd. Prír. 152: 22 (1983).<br><br>Heterotypic Synonyms: *Salvia pinardii* Boiss., Diagn. Pl. Orient. 12: 59 (1853); *Salvia rascheyana* Boiss., Diagn. Pl. Orient. 12: 58 (1853); *Salvia bodeana* Bunge, Labiat. Persic.: 42 (1873); *Salvia szovitsiana* Bunge, Labiat. Persic.: 43 (1873); *Schraderia acetabulosa* Pobed. in V.L.Komarov, Fl. URSS 21: 369 (1954).	*Salvia* s.s. clade I-D [5]	Cham.	W. Asia to Sinai	[19]
Salvia nemorosa L. - Sp. Pl. ed. 2: 35 (1762).	Homotypic Names: *Sclarea nemorosa* (L.) Mill., Gard. Dict. ed.8: nº6 (1768); *Sclarea punctata* Moench, Methodus: 374 (1794), nom. superfl; *Salvia* × *sylvestris* var. *nemorosa* (L.) Nyman, Consp. Fl. Eur.: 570 (1881).		Hemicr.	E. & C. Europe, W. & C. Asia	[19]
Salvia officinalis L. - Sp. Pl.: 23 (1753).	Heterotypic Synonyms: *Salvia officinalis* subsp. *minor* (C.C.Gmel.) Gams in G.Hegi, Ill. Fl. Mitt.-Eur. 5: 2483 (1927).	*Salvia* clade I [2], [3];	Cham.	SW. Germany to S. Europe	rosmarinic acid [36,37]

		Salvia s.s. clade I-D [4], [5]; *S. officinalis* clade [11]			
Salvia reuteriana Boiss. - Diagn. Pl. Orient. 5: 10 (1844)	-	[38]	Hemicr.	Iraq to Iran	rosmarinic acid [39]
Salvia sclarea L. - Sp. Pl.: 27 (1753)	Homotypic Names: *Sclarea vulgaris* Mill., Gard. Dict. ed.8: nº1 (1768); *Aethiopis sclarea* (L.) Opiz, Seznam: 11 (1852). Heterotypic Names: *Salvia haematodes* Scop., Fl. Carniol., ed.2, 1: 29 (1771), nom. illeg.; *Salvia coarctata* Vahl, Enum. Pl. Obs. 1: 253 (1804); *Salvia simsiana* Schult., Mant. 1: 210 (1822); *Salvia calostachya* Gand., Fl. Lyon.: 171 (1875); *Salvia sclarea* var. *calostachya* (Gand.) Nyman, Consp. Fl. Eur.: 569 (1881); *Salvia turkestanica* Noter, Rev. Hort. (Paris) 77: 502 (1905); *Salvia sclarea* var. *turkestaniana* Mottet, Rev. Hort. (Paris) 79: 135 (1907); *Salvia lucana* Cavara & Grande, Bull. Orto Bot. Regia Univ. Napoli 3: 436 (1913); *Salvia pamirica* Gand., Bull. Soc. Bot. France 60: 26 (1913); *Salvia altilabrosa* Pan, Trab. Mus. Ci. Nat., Ser. Bot. 14: 33 (1918).	*Salvia* clade I [2], [3]; *Salvia* s.s. clade I-C [4], [5]; *S. officinalis* clade [11]	Hemicr.	Medit. to C. Asia	aethiopinone, salvipisone, 1-oxo-aethiopinone, 1-oxo-ferruginol, carnosic acid, [40-45] 2a,3a-dihydroxy-urs-12-en-28-oic acid and 2a,3a-24-trihydroxy-urs-12-en-28-oic acid, b-sitosterol, stigmasterol, campesterol, oleanolic acid, ursolic acid [44]
Salvia tomentosa Mill. - Gard. Dict. ed. 8: n.º 2 (1768)	Homotypic Names: Salvia officinalis subsp. tomentosa (Mill.) P.Fourn., Quatre Fl. France: 835 (1938). Heterotypic Synonyms: *Salvia major* Garsault, Fig. Pl. Méd.: t. 510 a (1764), opus utique oppr.; *Salvia grandiflora* Etl., Salv.: 17 (1777); *Salvia rotundifolia* Vis., Mem. Reale Ist. Veneto Sci. 1: 42 (1842 publ. 1843), nom. illeg.; *Salvia fruticum* Vuk., Oesterr. Bot. Z. 30: 163 (1880); *Salvia nusairiensis* Post, Bull. Herb. Boissier 1: 406 (1893); *Salvia trigonocalyx* Woronow, Vestn. Tiflissk. Bot. Sada 22: 10 (1912); *Salvia officinalis* subsp. major Gams in G.Hegi, Ill. Fl. Mitt.-Eur. 5: 2483 (1927); *Salvia grandiflora* subsp. aegaea (Bornm.) Rech.f., Akad. Wiss. Wien, Math.-Naturwiss. Kl., Denkschr. 105: 519 (1943); *Salvia grandiflora* subsp. rotundifolia (Vis.) Rech.f., Akad. Wiss. Wien, Math.-Naturwiss. Kl., Denkschr. 105: 519 (1943); *Salvia brachystemon* Klokov, Fl. RSS Ukr. 9: 653 (1960).	*Salvia* s.s. clade I-D [5]	Cham. or nanophan.	SE. Europe to Transcaucasus	phenolic acids (gallic acid, protocatechuic acid, salicylic acid, chlorogenic acid, vanillic acid, caffeic acid, syringic acid, p-coumaric acid, sinapic acid, ferulic acid, cinnamic acid); flavonoids (myricetin, hesperidin, quercetin, luteolin, kaempferol) [46]

Salvia verticillata L. - Sp. Pl.: 26 (1753)	Homotypic Names: *Horminum verticillatum* (L.) Mill., Gard. Dict. ed. 8: n.º 3 (1768); *Covola verticillata* (L.) Medik., Philos. Bot. 2: 67 (1791); *Hemisphace verticillata* (L.) Opiz, Seznam: 50 (1852); *Sphacopsis verticillata* (L.) Briq., Lab. Alp. Mar.: 184 (1891)	*Salvia* clade I [2], [3]; *S. verticillata* group [5]; *S. officinalis* clade [11]	Hemicr.	C. Europe to Iran	[19]
Salvia virgata Jacq.	Heterotypic Names: *Salvia sibthorpii* Sm. in J.Sibthorp & J.E.Smith, Fl. Graec. Prodr. 1: 15 (1806); *Salvia campestris* M. Bieb., Fl. Taur-Caucas. 1: 20 (1808); *Salvia praecox* Loisel., Not. Fl. France: 6 (1810); *Salvia caduca* Vahl ex Hornem., Hort. Bot. Hafn. 1: 30 (1813); *Salvia mollis* J.Jacq., Ecl. Pl. Rar.: 56 (1813); *Salvia gigantea* Desf., Tabl. École Bot., ed. 2: 68 (1815); *Salvia affinis* Spreng. ex Steud., Nomencl. Bot. 1: 724 (1821); *Salvia hypanica* Andrz. in W.S.J.von Besser, Enum. Pl.: 3 (1821); *Salvia caucasica* Schrank, Syll. Pl. Nov. 2: 58 (1826); *Salvia rubra* Spreng., Syst. Veg. 4(2): 17 (1827); *Salvia garganica* Ten., Index Seminum (NAP, Neapolitano) 1829: 17 (1829); *Salvia amplexicaulis* Benth., Labiat. Gen. Spec.: 236 (1833), not validly publ.; *Salvia barrelieri* Benth., Labiat. Gen. Spec.: 235 (1833), not validly publ.; *Salvia bauhini* Benth., Labiat. Gen. Spec.: 235 (1833); *Salvia grandidentata* Ten., Index Seminum (NAP, Neapolitano) 1833: 15 (1833); *Salvia quercifolia* Benth., Labiat. Gen. Spec.: 235 (1833). Euriples rugosa Raf., Fl. Tellur. 3: 94 (1837); *Salvia nudicaulis* K.Koch, Linnaea 19: 24 (1846), nom. illeg.; *Salvia oblonga* K.Koch, Linnaea 19: 24 (1846); *Salvia virgata* var. *campestris* (M.Bieb.) Nyman, Consp. Fl. Eur.: 570 (1881); *Salvia virgata* var. *garganica* (Ten.) Nyman, Consp. Fl. Eur.: 570 (1881); *Salvia utilis* Braun ex Engl., Abh. Königl. Akad. Wiss. Berlin 1891: 367 (1892); *Salvia similata* Hausskn., Mitth. Thüring. Bot. Vereins, n.f., 11: 36 (1897); *Salvia virgata* f. *campestris* (M.Bieb.) E.Peter, Repert. Spec. Nov. Regni Veg. 39: 184 (1936); *Salvia extersa* Klokov, Fl. RSS Ukr. 9: 654 (1960); *Sclarea sibthorpii* (Sm.) Soják, Cas. Nár. Mus., Odd. Prír. 152: 22 (1983).	*Salvia* s.s. clade I-C [5]	Hemicr.	SE. Europe to C. Asia	rosmarinic acid [19,47]
Salvia viridis **L.**	Homotypic Names: *Horminum viride* (L.) Moench, Methodus: 377 (1794); *Ormilis viridis* (L.) Raf., Fl. Tellur. 3: 94 (1837); *Salvia horminum* var. *viridis* (L.) Caruel in	*Salvia* clade I [2], [3];	Ther.	S. Europe, NW. Africa, SW. Asia	rosmarinic acid, methyl rosmarinate, caffeic acid, protolithospermic acid,

	F.Parlatore, Fl. Ital. 6: 246 (1884); *Sclarea viridis* (L.) Soják, Cas. Nár. Mus., Odd. Prír. 152: 22 (1983). Heterotypic Names: *Salvia horminum* L., Sp. Pl.: 24 (1753); *Horminum sativum* Mill., Gard. Dict. ed.8: nº5 (1768); *Salvia spielmannii* Scop., Delic. Fl. Faun. Insubr. 3: 31 (1788); *Horminum coloratum* Moench, Methodus: 377 (1794); *Salvia comosa* Salisb., Prodr. Stirp. Chap. Allerton: 73 (1796); *Salvia colorata* Thore, Essai Chloris: 17 (1803), nom. illeg.; *Salvia truncata* Willd., Enum. Pl.: 34 (1809); *Salvia rosanii* Ten., Fl. Napol. 3: 22 (1824); *Flipanta ovata* Raf., Fl. Tellur. 3: 92 (1837), nom. superfl.; *Ormilis horminum* (L.) Raf., Fl. Tellur. 3: 94 (1837); *Salvia horminum* var. *angustifolia* Boiss., Fl. Orient. 4: 631 (1879); *Salvia viridis* var. *comata* Heldr., Fl. Céphalonie: 58 (1882); *Salvia viridis* var. *horminum* (L.) Batt. in J.A.Battandier & L.C.Trabut, Fl. Algérie, Dicot.: 685 (1890); *Salvia horminum* var. *hypoleuca* Briq., Lab. Alp. Mar.: 505 (1895); *Salvia horminum* var. *intermedia* Briq., Lab. Alp. Mar.: 503 (1895); *Salvia dolichorrhiza* Caball., Bol. Soc. Esp. Hist. Nat. 13: 238 (1913); *Salvia intercedens* Pobed. in V.L.Komarov, Fl. URSS 21: 657 (1954).	*Salvia* s.s. clade I-C [5]			salvianolic acid E, salvianolic acid F isomers, salvianolic acid J, hexoside derivatives of rosmarinic acid [48,49]
Salvia wagneriana Pol. - Linnaea 41: 591 (1878)	Heterotypic Synonyms: *Salvia tonduzii* Briq., Annuaire Conserv. Jard. Bot. Genève 2: 157 (1898); *Salvia albopileata* Epling, Ann. Missouri Bot. Gard. 27: 337 (1940).	Core *Calosphace*, *Fulgentes* clade, *Holvaya* subclade [17]	Nanophan.	SE. Mexico to C. America	rosmarinic acid [50]

References

1. Govaerts, R. World Checklist of Selected Plant Families. **2019**.
2. Walker, J.B.; Sytsma, K.J.; Treutlein, J.; Wink, M. *Salvia* (Lamiaceae) is not monophyletic: implications for the systematics, radiation, and ecological specializations of *Salvia* and tribe *Mentheae*. *American Journal of Botany* **2004**, *91*, 1115-1125.
3. Walker, J.B.; Sytsma, K.J. Staminal evolution in the genus *Salvia* (Lamiaceae): molecular phylogenetic evidence for multiple origins of the staminal lever. *Annals of Botany* **2007**, *100*, 375-391.
4. Dizkirici, A.; Celep, F.; Kansu, C.; Kahraman, A.; Dogan, M.; Kaya, Z. A molecular phylogeny of *Salvia euphratica* sensu lato (*Salvia* L., Lamiaceae) and its closely related species with a focus on the section *Hymenosphace*. *Plant Systematics and Evolution* **2015**, *301*, 2313-2323, doi:10.1007/s00606-015-1230-1.
5. Will, M.; Claßen-Bockhoff, R. Time to split *Salvia* sl (Lamiaceae) - New insights from Old World *Salvia* phylogeny. *Molecular Phylogenetics and Evolution* **2017**, *109*, 33-58.
6. Kuźma, Ł.; Kaiser, M.; Wysokińska, H. The production and antiprotozoal activity of abietane diterpenes in Salvia austriaca hairy roots grown in shake flasks and bioreactor. *Preparative Biochemistry & Biotechnology* **2017**, *47*, 58-66, doi:10.1080/10826068.2016.1168745.
7. Kuźma, Ł.; Wysokińska, H.; Różalski, M.; Krajewska, U.; Kisiel, W. An unusual taxodione derivative from hairy roots of Salvia austriaca. *Fitoterapia* **2012**, *83*, 770-773, doi:https://doi.org/10.1016/j.fitote.2012.03.006.

8. Kuźma, Ł.; Kisiel, W.; Królicka, A.; Wysokińska, H. Genetic transformation of Salvia austriaca by Agrobacterium rhizogenes and diterpenoid isolation. *Pharmazie* **2011**, *66*, 904-907.
9. Fraga, B.M.; Díaz, C.E.; Guadaño, A.; González-Coloma, A. Diterpenes from Salvia broussonetii Transformed Roots and Their Insecticidal Activity. *Journal of Agricultural and Food Chemistry* **2005**, *53*, 5200-5206, doi:10.1021/jf058045c.
10. Fraga, B.M.; Díaz, C.E.; López-Rodríguez, M. Two novel abietane dimers from transformed root cultures of Salvia broussonetii. *Tetrahedron Letters* **2014**, *55*, 877-879, doi:https://doi.org/10.1016/j.tetlet.2013.12.031.
11. Hu, G.X.; Takano, A.; Drew, B.T.; Liu, E.D.; Soltis, D.E.; Soltis, P.S.; Peng, H.; Xiang, C.L. Phylogeny and staminal evolution of *Salvia* (Lamiaceae, Nepetoideae) in East Asia. *Annals of Botany* **2018**, *122*, 649-668, doi:10.1093/aob/mcy104.
12. Wojciechowska, M.; Owczarek, A.; Kiss, A.K.; Grąbkowska, R.; Olszewska, M.A.; Grzegorczyk-Karolak, I. Establishment of hairy root cultures of Salvia bulleyana Diels for production of polyphenolic compounds. *Journal of Biotechnology* **2020**, *318*, 10-19, doi:https://doi.org/10.1016/j.jbiotec.2020.05.002.
13. Li, Q.Q.; Li, M.H.; Yuan, Q.J.; Cui, Z.H.; Huang, L.Q.; Xiao, P.G. Phylogenetic relationships of *Salvia* (Lamiaceae) in China: Evidence from DNA sequence datasets. *Journal of Systematics and Evolution* **2013**, *51*, 184-195.
14. Li, B.; Wang, B.; Li, H.; Peng, L.; Ru, M.; Liang, Z.; Yan, X.; Zhu, Y. Establishment of Salvia castanea Diels f. tomentosa Stib. hairy root cultures and the promotion of tanshinone accumulation and gene expression with Ag^{+}, methyl jasmonate, and yeast extract elicitation. *Protoplasma* **2016**, *253*, 87-100, doi:10.1007/s00709-015-0790-9.
15. Liu, L.; Yang, D.; Xing, B.; Zhang, H.; Liang, Z. Salvia castanea Hairy Roots are More Tolerant to Phosphate Deficiency than Salvia miltiorrhiza Hairy Roots Based on the Secondary Metabolism and Antioxidant Defenses. *Molecules* **2018**, *23*, 1132.
16. Jenks, A.A.; Walker, J.B.; Kim, S.-C. Phylogeny of New World *Salvia* subgenus *Calosphace* (Lamiaceae) based on cpDNA (psbA-trnH) and nrDNA (ITS) sequence data. *Journal of Plant Research* **2013**, *126*, 483-496.
17. Fragoso-Martínez, I.; Martínez-Gordillo, M.; Salazar, G.A.; Sazatornil, F.; Jenks, A.A.; Peña, M.d.R.G.; Barrera-Aveleida, G.; Benitez-Vieyra, S.; Magallón, S.; Cornejo-Tenorio, G. Phylogeny of the Neotropical sages (*Salvia* subg. Calosphace; Lamiaceae) and insights into pollinator and area shifts. *Plant Systematics and Evolution* **2018**, *304*, 43-55.
18. Fabriki-Ourang, S.; Yousefi-Azarkhanian, M. Genetic variability and relationships among Salvia ecotypes/species revealed by TRAP-CoRAP markers. *Biotechnology & Biotechnological Equipment* **2018**, *32*, 1486-1495, doi:10.1080/13102818.2018.1534555.
19. Norouzi, R.; Babalar, M.; Mirmasoumi, M. Investigation of hairy root induction in some Salvia L. species. *Nova Biologica Reperta* **2017**, *4*, 173-180, doi:10.21859/acadpub.nbr.4.2.173.
20. Takano, A.; Okada, H. Phylogenetic relationships among subgenera, species, and varieties of Japanese *Salvia* L. (Lamiaceae). *Journal of Plant Research* **2011**, *124*, 245-252.
21. Wei, T.; Gao, Y.; Deng, K.; Zhang, L.; Yang, M.; Liu, X.; Qi, C.; Wang, C.; Song, W.; Zhang, Y.; et al. Enhancement of tanshinone production in Salvia miltiorrhiza hairy root cultures by metabolic engineering. *Plant Methods* **2019**, *15*, 53, doi:10.1186/s13007-019-0439-3.
22. Wang, C.H.; Zheng, L.P.; Tian, H.; Wang, J.W. Synergistic effects of ultraviolet-B and methyl jasmonate on tanshinone biosynthesis in Salvia miltiorrhiza hairy roots. *J Photochem Photobiol B* **2016**, *159*, 93-100, doi:10.1016/j.jphotobiol.2016.01.012.
23. Xing, B.; Yang, D.; Guo, W.; Liang, Z.; Yan, X.; Zhu, Y.; Liu, Y. Ag+ as a more effective elicitor for production of tanshinones than phenolic acids in Salvia miltiorrhiza hairy roots. *Molecules (Basel, Switzerland)* **2014**, *20*, 309-324, doi:10.3390/molecules20010309.
24. Contreras, A.; Leroy, B.; Mariage, P.-A.; Wattiez, R. Proteomic analysis reveals novel insights into tanshinones biosynthesis in Salvia miltiorrhiza hairy roots. *Scientific Reports* **2019**, *9*, 5768, doi:10.1038/s41598-019-42164-3.
25. Wang, Q.J.; Zheng, L.P.; Yuan, H.Y.; Wang, J. Propagation of Salvia miltiorrhiza from hairy root explants via somatic embryogenesis and tanshinone content in obtained plants. *Industrial Crops and Products* **2013**, *50*, 648-653, doi:https://doi.org/10.1016/j.indcrop.2013.08.031.
26. Hao, X.; Shi, M.; Cui, L.; Xu, C.; Zhang, Y.; Kai, G. Effects of methyl jasmonate and salicylic acid on tanshinone production and biosynthetic gene expression in transgenic Salvia miltiorrhiza hairy roots. *Biotechnol Appl Biochem* **2015**, *62*, 24-31, doi:10.1002/bab.1236.

27. Wu, J.-Y.; Shi, M. Ultrahigh diterpenoid tanshinone production through repeated osmotic stress and elicitor stimulation in fed-batch culture of Salvia miltiorrhiza hairy roots. *Applied Microbiology and Biotechnology* **2008**, *78*, 441-448, doi:10.1007/s00253-007-1332-y.
28. Cheng, Q.; He, Y.; Li, G.; Liu, Y.; Gao, W.; Huang, L. Effects of Combined Elicitors on Tanshinone Metabolic Profiling and SmCPS Expression in Salvia miltiorrhiza Hairy Root Cultures. *Molecules* **2013**, *18*, 7473-7485.
29. Shi, M.; Kwok, K.W.; Wu, J.Y. Enhancement of tanshinone production in Salvia miltiorrhiza Bunge (red or Chinese sage) hairy-root culture by hyperosmotic stress and yeast elicitor. *Biotechnol Appl Biochem* **2007**, *46*, 191-196, doi:10.1042/ba20060147.
30. Liang, Z.-S.; Yang, D.-F.; Liang, X.; Zhang, Y.-J.; Liu, Y.; Liu, F.-H. Roles of reactive oxygen species in methyl jasmonate and nitric oxide-induced tanshinone production in Salvia miltiorrhiza hairy roots. *Plant Cell Reports* **2012**, *31*, 873-883, doi:10.1007/s00299-011-1208-6.
31. Pei, T.; Ma, P.; Ding, K.; Liu, S.; Jia, Y.; Ru, M.; Dong, J.; Liang, Z. SmJAZ8 acts as a core repressor regulating JA-induced biosynthesis of salvianolic acids and tanshinones in Salvia miltiorrhiza hairy roots. *Journal of Experimental Botany* **2017**, *69*, 1663-1678, doi:10.1093/jxb/erx484.
32. Zhi, B.H.; Alfermann, A.W. Diterpenoid production in hairy root cultures of Salvia miltiorrhiza. *Phytochemistry* **1993**, *32*, 699-703, doi:https://doi.org/10.1016/S0031-9422(00)95156-2.
33. Liu, L.; Yang, D.; Liang, T.; Zhang, H.; He, Z.; Liang, Z. Phosphate starvation promoted the accumulation of phenolic acids by inducing the key enzyme genes in Salvia miltiorrhiza hairy roots. *Plant Cell Rep* **2016**, *35*, 1933-1942, doi:10.1007/s00299-016-2007-x.
34. Zhou, Z.; Tan, H.; Li, Q.; Chen, J.; Gao, S.; Wang, Y.; Chen, W.; Zhang, L. CRISPR/Cas9-mediated efficient targeted mutagenesis of RAS in Salvia miltiorrhiza. *Phytochemistry* **2018**, *148*, 63-70, doi:10.1016/j.phytochem.2018.01.015.
35. Shi, M.; Luo, X.; Ju, G.; Li, L.; Huang, S.; Zhang, T.; Wang, H.; Kai, G. Enhanced Diterpene Tanshinone Accumulation and Bioactivity of Transgenic Salvia miltiorrhiza Hairy Roots by Pathway Engineering. *Journal of Agricultural and Food Chemistry* **2016**, *64*, 2523-2530, doi:10.1021/acs.jafc.5b04697.
36. Grzegorczyk, I.; Królicka, A.; Wysokińska, H. Establishment of Salvia officinalis L. hairy root cultures for the production of rosmarinic acid. *Z Naturforsch C J Biosci* **2006**, *61*, 351-356, doi:10.1515/znc-2006-5-609.
37. Grzegorczyk, I.; Wysokiñska, H. Antioxidant compounds in *Salvia officinalis* L. shoot and hairy root cultures in the nutrient sprinkle bioreactor. *Acta Societatis Botanicorum Poloniae* **2010**, *79*, 7-10.
38. Ranjbar, M.; Pakatchi, A.; Babataheri, Z. Chromosome number evolution, biogeography and phylogenetic relationships in Salvia (Lamiaceae). *Webbia* **2015**, *70*, 293-312, doi:10.1080/00837792.2015.1057982.
39. NOROUZI, R.; BABALAR, M.; MIRMASOUMI, M.; HADIAN, J. PRODUCTION OF ROSMARINIC ACID IN HAIRY ROOT CULTURES OF SALVIA REUTERANA. *JOURNAL OF AGRICULTURAL BIOTECHNOLOGY* **2016**, *8*, -.
40. Vaccaro, M.C.; Mariaevelina, A.; Malafronte, N.; De Tommasi, N.; Leone, A. Increasing the synthesis of bioactive abietane diterpenes in Salvia sclarea hairy roots by elicited transcriptional reprogramming. *Plant Cell Reports* **2017**, *36*, 375-386, doi:10.1007/s00299-016-2076-x.
41. Kuźma, Ł.; Bruchajzer, E.; Wysokińska, H. Methyl jasmonate effect on diterpenoid accumulation in Salvia sclarea hairy root culture in shake flasks and sprinkle bioreactor. *Enzyme and Microbial Technology* **2009**, *44*, 406-410, doi:https://doi.org/10.1016/j.enzmictec.2009.01.005.
42. Kuźma, L.; Bruchajzer, E.; Wysokińska, H. Diterpenoid production in hairy root culture of Salvia sclarea L. *Z Naturforsch C J Biosci* **2008**, *63*, 621-624, doi:10.1515/znc-2008-7-827.
43. Walencka, E.; Rozalska, S.; Wysokinska, H.; Rozalski, M.; Kuzma, L.; Rozalska, B. Salvipisone and aethiopinone from Salvia sclarea hairy roots modulate staphylococcal antibiotic resistance and express anti-biofilm activity. *Planta Med* **2007**, *73*, 545-551, doi:10.1055/s-2007-967179.
44. Kuźma, Ł.; Skrzypek, Z.; Wysokińska, H. Diterpenoids and triterpenoids in hairy roots of Salvia sclarea. *Plant Cell, Tissue and Organ Culture* **2006**, *84*, 171-179, doi:10.1007/s11240-005-9018-6.
45. Kuźma, Ł.; Różalski, M.; Walencka, E.; Różalska, B.; Wysokińska, H. Antimicrobial activity of diterpenoids from hairy roots of Salvia sclarea L.: Salvipisone as a potential anti-biofilm agent active against antibiotic resistant Staphylococci. *Phytomedicine* **2007**, *14*, 31-35, doi:10.1016/j.phymed.2005.10.008.
46. Marchev, A.; Georgiev, V.; Ivanov, I.; Badjakov, I.; Pavlov, A. Two-phase temporary immersion system for Agrobacterium rhizogenes genetic transformation of sage (Salvia tomentosa Mill.). *Biotechnol Lett* **2011**, *33*, 1873-1878, doi:10.1007/s10529-011-0625-5.

47. Dowom, S.A.; Abrishamchi, P.; Radijabian, T.; Salami, S.A. Methyl jasmonate enanches the accumulation of phenolic acids in Salvia virgata Jacq. hairy root cultures. In Proceedings of the 7th National Congress on Medicinal Plants, Shiraz, Iran, 12-14th May 2018, 2018; p. 314.
48. Grzegorczyk-Karolak, I.; Kuźma, Ł.; Skała, E.; Kiss, A.K. Hairy root cultures of Salvia viridis L. for production of polyphenolic compounds. *Industrial Crops and Products* **2018**, *117*, 235-244, doi:https://doi.org/10.1016/j.indcrop.2018.03.014.
49. Grzegorczyk-Karolak, I. Optimization of culture conditions and cultivation phase for the growth of Salvia viridis transformed roots and polyphenolic compound production. *Plant Cell, Tissue and Organ Culture (PCTOC)* **2020**, *142*, 571-581, doi:10.1007/s11240-020-01883-6.
50. Ruffoni, B.; Bertoli, A.; Pistelli, L.; Pistelli, L. Micropropagation of *Salvia wagneriana* Polak and hairy root cultures with rosmarinic acid production. *Natural Product Research* **2016**, *30*, 2538-2544, doi:10.1080/14786419.2015.1120725.
